# Effect of erythritol on microbial ecology of *in vitro* gingivitis biofilms

**DOI:** 10.1080/20002297.2017.1337477

**Published:** 2017-06-22

**Authors:** Marleen Marga Janus, Catherine Minke Charlotte Volgenant, Bernd Willem Brandt, Mark Johannes Buijs, Bart Jan Frederik Keijser, Wim Crielaard, Egija Zaura, Bastiaan Philip Krom

**Affiliations:** ^a^ Department of Preventive Dentistry, Academic Centre for Dentistry Amsterdam, University of Amsterdam and Vrije Universiteit Amsterdam, Amsterdam, The Netherlands; ^b^ Top Institute Food and Nutrition, Wageningen, The Netherlands; ^c^ Microbiology and Systems Biology, TNO Earth, Environmental and Life Sciences, Zeist, The Netherlands

**Keywords:** *In vitro* oral biofilms, gingivitis, microbiome, polyol, biofilm maturation, biofilm phenotype

## Abstract

Gingivitis is one of the most common oral infections in humans. While sugar alcohols such as erythritol are suggested to have caries-preventive properties, it may also have beneficial effects in prevention of gingivitis by preventing maturation of oral biofilms. The aim of this study was to assess the effect of erythritol on the microbial ecology and the gingivitis phenotype of oral microcosms. Biofilms were inoculated with stimulated saliva from 20 healthy donors and grown in a gingivitis model in the continuous presence of 0 (control group), 5, and 10% erythritol. After 9 days of growth, biofilm formation, protease activity (gingivitis phenotype), and microbial profile analyses were performed. Biofilm growth was significantly reduced in the presence of erythritol, and this effect was dose dependent. Protease activity and the Shannon diversity index of the microbial profiles of the biofilms were significantly lower when erythritol was present. Microbial profile analysis revealed that presence of erythritol induced a compositional shift from periodontitis- and gingivitis-related taxa toward early colonizers. The results of this study suggest that erythritol suppresses maturation of the biofilms toward unhealthy composition. The gingivitis phenotype was suppressed and biofilm formation was reduced in the presence of erythritol. Therefore, it is concluded that erythritol may contribute to a healthy oral ecosystem *in vitro*.

## Introduction

In the oral cavity, two major plaque-induced diseases occur: dental caries and gingivitis. Caries is a consequence of frequent consumption of dietary fermentable sugars. This leads to frequent and prolonged periods of acid production by microbes residing in dental plaque. Acid production by dental plaque can result in dissolving the hard tissues in the oral cavity and tooth decay [1]. Gingivitis is inflammation of the gums. When left untreated, prolonged periods of inflammation can ultimately lead to periodontitis, which is the progressive loss of the alveolar bone surrounding the teeth [2–4].

Oral diseases can be prevented by good oral hygiene, including the use of fluoride, combined with a lower frequency of dietary fermentable sugar consumption [5,6]. In addition, replacement of dietary fermentable sugars by artificial sweeteners, such as sugar alcohols, has been suggested to have a caries-preventive effect [7]. Polyol sweeteners are not metabolized by most oral bacteria [8], thus significantly reducing the acid production by dental plaque. One example of such a sweetener is erythritol, a polyol that is not metabolized in the human body [9]. Compared to other sweeteners such as xylitol, erythritol induces only low gastrointestinal responses and does not induce diarrhea. At low doses, no adverse effects have been reported [10], making it an attractive food additive. Erythritol has been shown to have potential caries-reducing properties [11,12]. It reduces the abundance of *Streptococcus mutans*, significant acid-producing species, in plaque, as well as total plaque accumulation after 6 months’ consumption [13], while plaque levels of propionic acid, acetic acid, and lactic acid were reduced after 3 years of erythritol consumption [14].

Few studies have tested whether erythritol is also beneficial for gingival health. Gingivitis develops in response to increased plaque accumulation [15]. Specific bacterial species, most notably *Porphyromonas gingivalis*, have been linked to gingivitis and periodontitis [16]. Since these are late-colonizing species, timely and thorough removal of dental plaque is the main gingivitis preventive therapy [15]. This keeps the plaque buildup under critical levels and prevents changes in species composition related to maturation of dental plaque. Failure to remove plaque adequately can result in progression toward periodontitis.

Hashino et al. found that erythritol reduces the abundance of the periodontitis-related bacterium *P. gingivalis* in dual-species biofilms with *Streptococcus gordonii* [17]. This study suggested that DNA/RNA synthesis is decreased in both *P. gingivalis* and *S. gordonii* when 10% erythritol is present, resulting in a retarded bacterial growth. The same study showed that amino sugar and nucleotide sugar metabolites were decreased when 10% erythritol was present. This probably retards extracellular matrix biosynthesis and partly explains the altered microstructure of the dual-species biofilms found by Hashino et al. [17].

To the authors’ knowledge, no multispecies biofilm studies exist that report the effect of erythritol on gingivitis-related biofilms. Therefore, this study investigated the effect of erythritol on the ecology and the gingivitis phenotype of *in vitro* oral microcosm biofilms.

## Materials and methods

### Saliva collection

The saliva samples used in this study were derived from participants of a previous cohort study [18]. This cohort study was conducted in accordance with the ethical principles of the 64th WMA Declaration of Helsinki (October 2013, Brazil) and the Medical Research Involving Human Subjects Act, approximating Good Clinical Practice (CPMP/ICH/135/95) guidelines. The clinical trial was approved by the Medical Ethical Committee of the VU Medical Center (2014.505) and registered at the public trial register of the Central Committee on Research Involving Human Subjects under number NL51111.029.14. Volunteers received oral and written information about the study at the screening session, and all participants of the clinical study signed the informed consent. The inclusion and exclusion criteria have been described previously [18]. The level of gingival bleeding was assessed using the bleeding on marginal probing, as described previously [19], in a half mouth randomized contralateral model [18].

From 20 of the systemically and orally healthy donors of the cohort study, baseline stimulated saliva samples were collected and used for the present study. The participants did not perform any oral hygiene measures for at least 5 h, and they did not drink or eat in the 2 h prior to saliva donation. Stimulated saliva was collected and stored, as described previously [20]. Inocula for the biofilm model were created by mixing stimulated saliva of a single volunteer at a 1:50 ratio with fresh biofilm growth medium, as described below.

### Biofilm formation

The inocula were added to the *in vitro* Amsterdam Active Attachment Model (AAA-model) [21] assembled with 12 mm glass coverslips (Menzel, Braunsweig, Germany) to form biofilms. Biofilms were grown in buffered semi-defined McBain medium supplemented with 10% fetal calf serum (FCS; Sigma–Aldrich, Munich, Germany) to promote gingivitis-related phenotypes [20]. For each donor, four biofilms per erythritol concentration were inoculated for 8 h in growth medium. After 8 h of inoculation, the medium was refreshed, and biofilms were grown anaerobically in continuous presence of 0, 5, or 10% (w/v) erythritol (Zerose® Eryhritol; Cargill, Vilvoorde, Belgium) for 9 days at 37°C. The medium, containing serum and 0, 5, or 10% erythritol, was refreshed once a day. These conditions allow for the development of matured biofilms expressing high protease activity, indicative of a gingivitis or periodontitis phenotype [20].

### Biofilm quantification

Total anaerobic colony forming units (CFUs) for each individual biofilm were determined to estimate the amount of biofilm [21]. The 9-day-old biofilms were dispersed in phosphate-buffered saline by sonication [20], serially diluted, and plated on tryptic soy agar blood agar plates for total counts.

### Protease activity

Gingivitis and periodontitis are related to increased total and specific protease activity [3]. Gingipains are *P. gingivalis*–specific proteases and important virulence factors. Clinically, gingipain activity is related to periodontitis [22]. Total and specific protease activity was measured, as described previously [20,22], using a fluorescence resonance energy transfer (FRET) assay. For total protease activity, the FRET-probe PEK-054 ([FITC]-NleKKKKVLPIQLNAATDK-[KDbc]) [23] was used, and specific protease activity was measured using the FRET-probe BikKam15 ([FITC]-Phe-Arg-[KDbc]) [22]. The final concentration of the probe was 16 µM, and fluorescence (excitation at 485 nm and emission at 530 nm) was measured every 2 min for 2 h. Protease activity was defined in relative fluorescence per minute (RF/min) determined from the initial linear part of the curve.

### DNA isolation and microbiome analysis

All individual biofilms (i.e. four per donor per erythritol concentration) were used for microbiome analysis. Total DNA was isolated and purified, as previously described [20]. Bacterial DNA concentration was determined by quantitative polymerase chain reaction (qPCR) using a universal primer-probe set targeting the 16S rRNA gene [24]. Next, 1 ng of DNA was used to amplify the V4 hypervariable region of the 16S rRNA gene, as described previously [25], except that 33 amplification cycles were performed. The amplicons were pooled equimolarly and purified from agarose gel (Illustra™, GE Healthcare, Little Chalfont, United Kingdom). Paired-end reads of 251 bp were generated by paired-end sequencing of the amplicons using the Illumina MiSeq platform and Illumina MiSeq reagent kit V2 (Illumina, Inc., San Diego, CA) at the VUmc Cancer Center Amsterdam (Amsterdam, The Netherlands). The sequence data were processed, as described previously [26], albeit with a maximum of 25 mismatches in the overlap. In addition, a species name was assigned to the representative (most abundant) sequence of the operational taxonomic unit (OTU) based on the Human Oral Microbiome Database (HOMD) [27]. First, BLAST was used to obtain the top 20 matches in the HOMD database (BLAST on homd.org; HOMD 16S rRNA RefSeq version 14.5 database). A HOMD taxonomy was assigned only if the similarity was ≥97% and if the entire query sequence was aligned. In case of tied top hits (i.e. same percentage similarity), the (species) names were combined into the taxonomy assigned.

### Data normalization and statistical analysis

The OTUs were randomly subsampled at 5,200 reads/sample, and the median abundance of each OTU in the quadruple biofilms was calculated for each donor and condition. PAST software v3.01 [28] was used to calculate the species richness and the Shannon diversity index. Significance of the differences in species richness and diversity was calculated using a Friedman test in SPSS Statistics for Windows v23.0.0.2 (IBM Corp., Armonk, NY).

The OTU-table was log_2_ transformed, and PAST was used to ordinate the data by principal component analysis (PCA) into two dimensions. To calculate the significance of the compositional differences between 0, 5, and 10% erythritol biofilms, one-way permutational multivariate analysis of variance was performed on the Bray–Curtis Similarity index. Groups were considered statistically different at *p *< 0.05.

To identify the OTUs that differ in relative abundance between 0, 5, or 10% erythritol, linear discriminant analysis effect size (LEfSe) [29] was used in the one-against-all modus. The alpha values were kept at the default of 0.05, and the LDA threshold was set to 3.5.

## Results

### Biofilm formation

**Figure 1.**
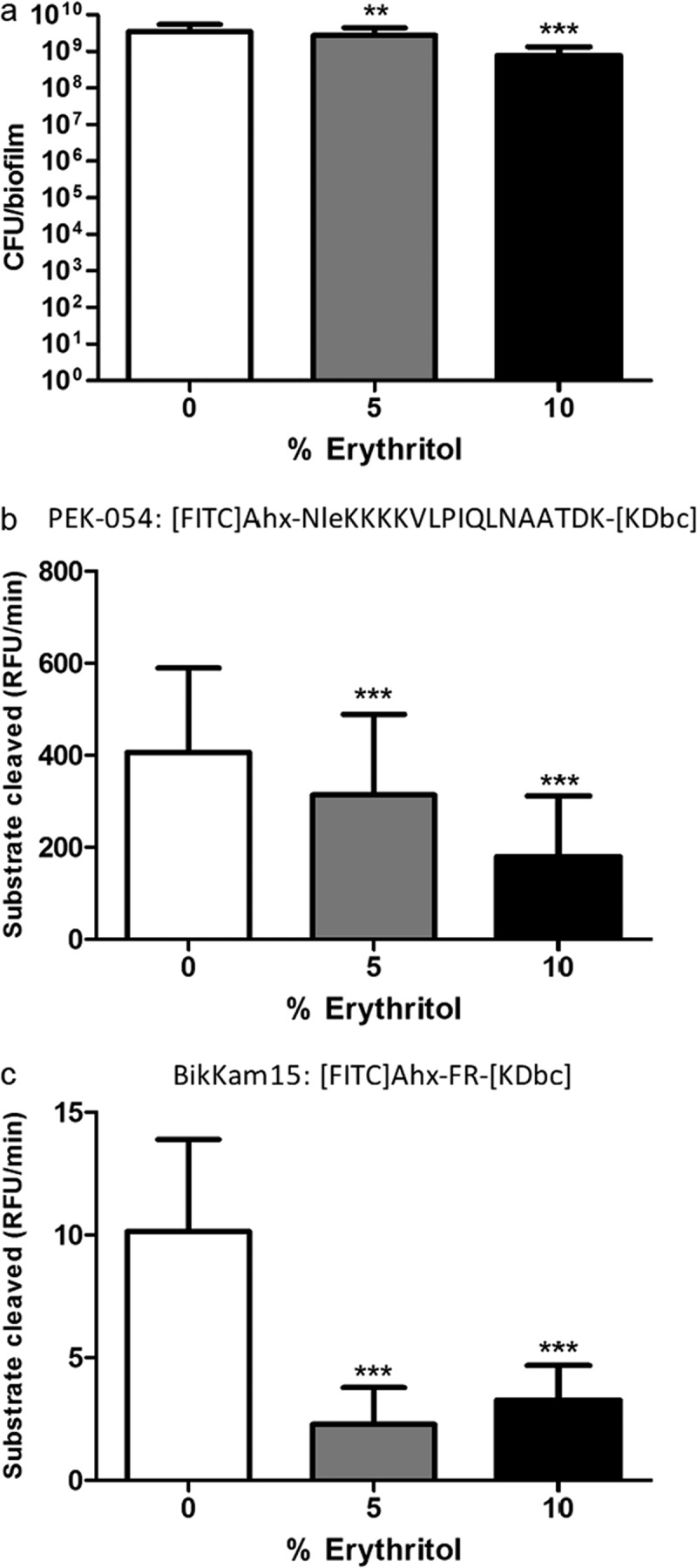
Phenotypes of the biofilms grown in presence of different concentrations of erythritol: (a) colony forming unit (CFU) counts, (b) total protease activity measured using fluorescence resonance energy transfer (FRET) probe PEK-054 (expressed as RFU/min), (c) specific protease activity measured using FRET-probe BikKam15 (expressed as RFU/min). Statistical significance compared to the control is indicated (**p < 0.01; ***p < 0.001).

Stimulated saliva of 20 orally and systemically healthy donors was used to inoculate the AAA-model. For every donor, quadruple biofilms were grown anaerobically for 9 days in the presence of 0, 5, or 10% erythritol. Biofilm density showed an erythritol-dependent decline. When grown in the presence of 5% erythritol, CFUs of the biofilm were significantly less (*p* < 0.01) compared to the control. When cultivated in the presence of 10% erythritol, the number of CFUs was even lower (Figure 1a). The bacterial density of biofilms grown in the presence of 10% erythritol was on average 80% lower than that of the control biofilms (0.5 log_10_ difference).

### Protease activity

Total protease activity and specific activity were measured to evaluate the effect of erythritol on gingivitis phenotype of the biofilms. Total as well as specific protease activity was lower in biofilms grown in the presence of erythritol compared to the control biofilms (Figure 1b and c). Total protease activity fell from 406 RFU/min (*SD* = 183) on average for the control biofilms to 314 RFU/min (*SD* = 175) in 5% erythritol biofilms, and 179 RFU/min (*SD* = 132) in 10% erythritol biofilms, showing a dose-dependent reduction. Specific protease activity was reduced from 10.1 RFU/min (*SD* = 3.8) in the control biofilms to 2.3 RFU/min (*SD* = 1.5) in the 5% erythritol biofilms. Biofilms grown in the presence of 10% erythritol, however, had a small but statistically significant higher specific protease activity (3.3 RFU/min; *SD* = 1.4) compared to the 5% erythritol biofilms. This slight increase in specific protease activity in 10% compared to 5% erythritol biofilms was observed in 17/20 individual donors (data not shown). Nonetheless, the specific protease activity in the biofilms grown in the presence of 10% erythritol was low compared to the control samples.

### Microbial composition

To evaluate the effect of erythritol on the microbial composition of the biofilms, the bacterial DNA was analysed using 16S rDNA sequencing. The complete subsampled OTU list is available as Supplementary Table S1.

**Figure 2.**
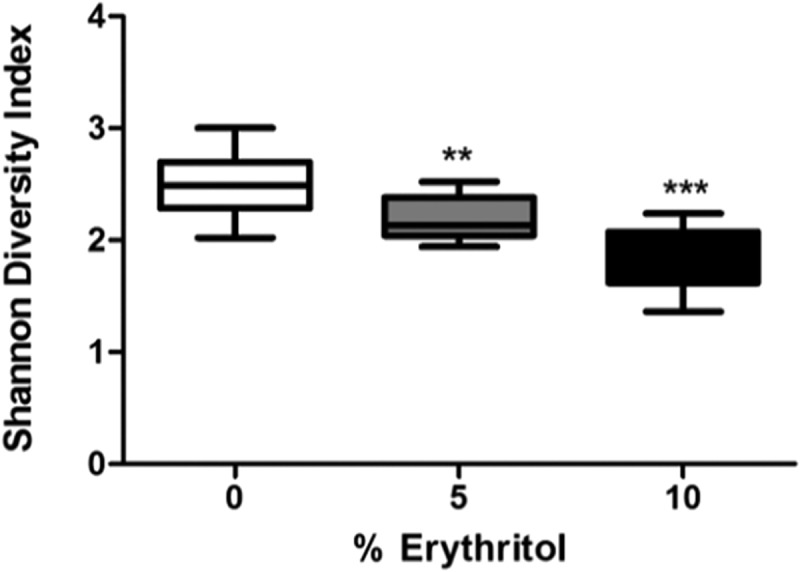
Shannon diversity index of the biofilms grown in presence of different concentrations of erythritol. Statistical significance compared to the control is indicated (**p < 0.01; ***p < 0.001).

**Figure 3.**
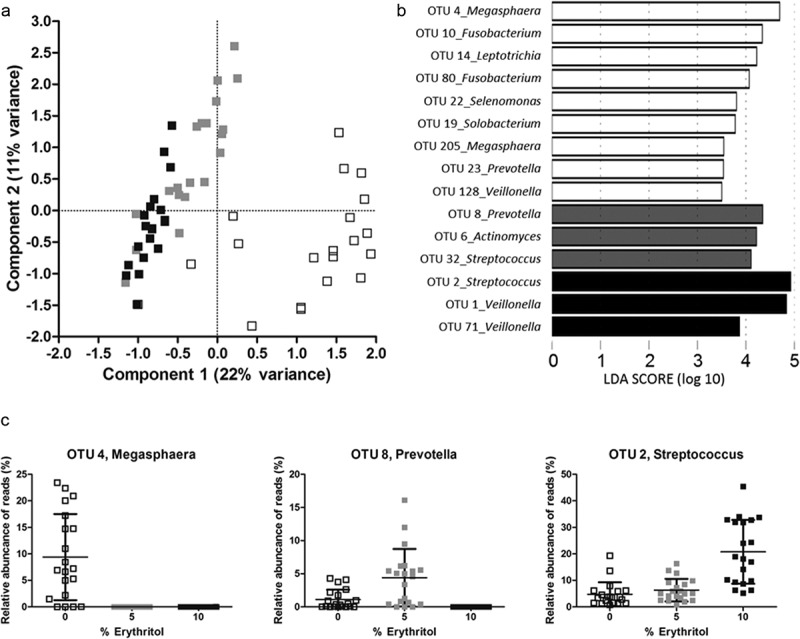
Microbiome analysis of biofilms grown in the presence of 0, 5, or 10% erythritol. (a) Principal component analysis plot of biofilms grown in the presence of 0 (□), 5 ( ), or 10% (∎∎) erythritol. The data were randomly subsampled and log_2_-transformed. (b) Operational taxonomic units (OTUs) that differentiate most between 0 (white bars), 5 (gray bars), or 10% (black bars) erythritol, ranked by the effect size in linear discriminant analysis effect size (LEfSe). (c) Boxplots of the relative abundance of the most prominent biomarker detected with LEfSe for each condition: OTU4 for 0%, OTU8 for 5%, and OTU2 for 10%.

The species richness of the biofilms was 73 ± 14 OTUs for 0% erythritol, 62 ± 12 for 5% erythritol, and 49 ± 9 for 10% erythritol. These differences were statistically significant. In line with this, the Shannon diversity index of the biofilms was significantly lower (χ^2^ = 39.5; *p* < 0.001) if erythritol was present: 2.5 ± 0.3 for the control biofilms versus 2.2 ± 0.2 and 1.8 ± 0.2 for 5 and 10% erythritol biofilms, respectively (Figure 2). The species composition was clearly different between the three groups (*p* < 0.001; *F* = 10.8). This is visualized in the PCA plot (Figure 3a) where the biofilms clustered based on the concentration of erythritol present during growth. To identify the OTUs that showed significantly different abundance under the different erythritol concentrations (biomarkers), LEfSe [29] was used. A total of 15 OTUs were significantly different in relative abundance between at least two of the three groups (Figure 3b). The relative abundance of the OTUs detected by LEfSe is plotted in Supplementary Figure S1. The relative abundance of the most prominent biomarker for each condition is shown in Figure 3c.

LEfSe indicated that the control biofilms, grown for 9 days in the presence of FCS, contained more reads classified as *Megasphaera* (OTU4, OTU205), *Fusobacterium* (OTU10, OTU80), *Leptotrichia* (OTU14), *Selenomonas* (OTU22), and *Solobacterium* (OTU19) compared to both erythritol conditions. In biofilms from some volunteers, *Prevotella* (OTU23) was exclusively present in 0% erythritol biofilms, while in other volunteers, this OTU was not present at all. Biofilms grown in the presence of 5% erythritol contained more *Prevotella* (OTU8), *Actinomyces* (OTU6), and *Streptococcus* (OTU32) compared to 0 and 10% biofilms. Biofilms grown in the presence of 10% erythritol contained more *Streptococcus* (OTU2) compared to the other conditions.

**Figure 4.**
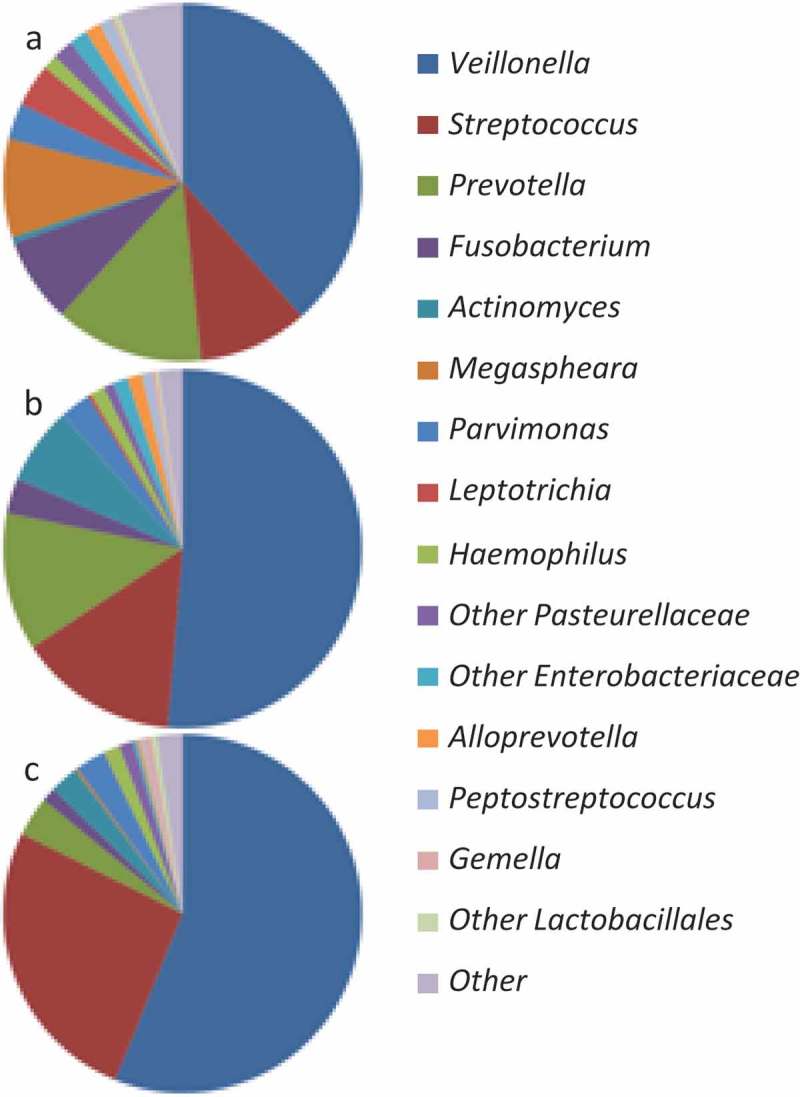
Average relative abundance of 20 of the most abundant genera (remaining genera are grouped as ‘other’) in: (a) 0% erythritol biofilms, (b) 5% erythritol biofilms, and (c) 10% erythritol biofilms.

Different OTUs of *Veillonella* were identified as biomarkers for both the control biofilms and the 10% erythritol biofilms. Control biofilms showed higher abundance of OTU128, and 10% erythritol biofilms contained more of OTU1 and OTU71. Although some *Veillonella* OTUs were more abundant in the control biofilms, the total abundance of the genus significantly increased when erythritol was present (38% abundance for control biofilms, 51% abundance for 5% erythritol biofilms, and 56% abundance for 10% erythritol biofilms) (Figure 4).

## Discussion

In this study, biofilms grown in the presence of erythritol showed a significantly different bacterial species composition and were less diverse compared to the control biofilms. Erythritol also reduced biofilm formation and both total and specific protease activity of the biofilms.

These results show that presence of erythritol reduced biofilm formation of *in vitro* gingivitis-phenotype biofilms. This is in line with a previous study, where growth of several *Streptococcus* species, including clinical oral isolates, was reduced when erythritol was present [30]. Several clinical trials investigating the caries-reducing effect of erythritol described a lower amount of plaque in the erythritol test group [13,14]. Although the reduction in biofilm formation in this study was statistically significant, the biofilms grown in the presence of erythritol still consisted of high numbers of viable microbes. The biological relevance of this reduction is uncertain. The 9-day-old biofilms, consisting of cell counts >10^8^, still formed a thick layer of plaque in the biofilm model.

The growth conditions of the biofilms were chosen to induce a gingivitis phenotype [20]. The serum added to the growth medium mimics crevicular fluid, stimulating development of a proteolytic microbiome. Together with the proteolytic stimulation, 9 days of growth results in a mature gingivitis biofilm, as observed for the control biofilms. The addition of erythritol significantly reduced the proteolytic activity of the biofilms, indicating a reduction of the gingivitis phenotype. This is in line with a previous study showing that erythritol inhibited gingipain activity of *P. gingivalis* [17]. Reduced total protease activity in presence of erythritol could indicate that the dynamic transition from saccharolytic, young plaque to proteolytic, mature plaque is inhibited by erythritol. Reduced specific proteolytic activity furthermore indicates reduction of an important virulence factor related to periodontitis.

Besides the reduction of biofilm growth and protease activity, bacterial diversity decreased with increasing erythritol concentrations. Plaque from periodontitis patients has been reported to have a higher microbial diversity than plaque from periodontally healthy patients has [31,32]. This adds up to the idea that erythritol contributes to preserving a healthy oral ecology.

Ecological analysis indicates that presence of erythritol significantly affects the bacterial species composition. Control biofilms contained *Megasphaera*, *Solobacterium, Selenomonas*, and *Leptotrichia*, which were all absent when erythritol was present. All these genera are associated with periodontal disease [33–35] or gingivitis [36]. Additionally, *Fusobacterium* was present in higher abundance in the control biofilms. This is a well-known ‘bridging’ organism that is able to co-aggregate with periodontal pathogens to form a mature biofilm [1]. Reduction or depletion of these genera supports the conclusion of Hashino et al. that erythritol can be used to prevent periodontal diseases [17].

Abundance of *Prevotella* was high in both the control and 5% erythritol biofilms, and it decreased when 10% of erythritol was present. *Prevotella* has been previously associated with dental plaque obtained from gingivitis patients [36], suggesting that the effect of erythritol on the microbial ecology is concentration dependent. *Streptococcus* and *Veillonella* species were more abundant in biofilms grown in the presence of erythritol, and they seem to increase with higher erythritol concentrations. Both species are identified as early colonizers of dental biofilms [1], indicating that erythritol inhibits development toward a mature biofilm.

The exact mechanism that is responsible for inhibition of biofilm maturation is not yet understood. Hashino et al. showed that the presence of erythritol retards growth and extracellular matrix biosynthesis [17]. Although this might partly explain the reduction in biofilm formation observed in this study, it does not explain the shift in microbial ecology.

One possible explanation of this shift is the decrease in protease activity when erythritol was present. Inhibition of the protease activity might reduce the availability of dipeptides and amino acids, specifically inhibiting growth of proteolytic microbes. This is in line with the Hashino study, where relative abundance of *P. gingivalis* decreased in dual-species biofilms with *S. gordonii*, showing that *S. gordonii* is less sensitive to erythritol compared to *P. gingivalis*. Differential sensitivity to erythritol might explain ecological shifts, as observed in the present study.

Summarizing, erythritol induces a compositional shift from periodontitis- and gingivitis-related species toward an ecology dominated by the traditional early colonizers. This suggests that erythritol suppresses the maturation of oral biofilms and prevents the development of an unhealthy ecology. Additionally, the gingivitis phenotype was suppressed and biofilm formation reduced in the presence of erythritol. Therefore, from these results, it is concluded that erythritol may contribute to a healthy oral ecosystem.

## Supplementary Material

Supplemental_data.zip

## References

[1] Kolenbrander PE, Palmer RJ Jr., Periasamy S, et al Oral multispecies biofilm development and the key role of cell-cell distance. Nat Rev Microbiol. 2010;8(7):471–8. 20514044 10.1038/nrmicro2381

[2] Kistler JO, Booth V, Bradshaw DJ, et al Bacterial community development in experimental gingivitis. Plos One. 2013;8(8):e71227. 23967169 10.1371/journal.pone.0071227 PMC3743832

[3] Marsh PD. Microbial ecology of dental plaque and its significance in health and disease. Adv Dent Res. 1994;8(2):263–271. 7865085 10.1177/08959374940080022001

[4] Sanz M, Van Winkelhoff AJ Working group 1 of Seventh European Workshop on Periodontal infections: understanding the complexity–consensus of the Seventh European Workshop on Periodontology. J Clin Periodontol. 2011;38 Suppl 11:3–6. 21323698 10.1111/j.1600-051X.2010.01681.x

[5] Rugg-Gunn A Dental caries: strategies to control this preventable disease. Acta Med Acad. 2013;42(2):117–130. 24308392 10.5644/ama2006-124.80

[6] ten Cate JM. Current concepts on the theories of the mechanism of action of fluoride. Acta Odontol Scand. 1999;57(6):325–329. 10777135 10.1080/000163599428562

[7] Van Loveren C Sugar alcohols: what is the evidence for caries-preventive and caries-therapeutic effects? Caries Res. 2004;38(3):286–293. 15153702 10.1159/000077768

[8] Bradshaw DJ, Marsh PD Effect of sugar alcohols on the composition and metabolism of a mixed culture of oral bacteria grown in a chemostat. Caries Res. 1994;28(4):251–256. 8069881 10.1159/000261977

[9] Hiele M, Ghoos Y, Rutgeerts P, et al Metabolism of erythritol in humans: comparison with glucose and lactitol. Br J Nutr. 1993;69(1):169–176. 8457525 10.1079/bjn19930019

[10] Storey D, Lee A, Bornet F, et al Gastrointestinal tolerance of erythritol and xylitol ingested in a liquid. Eur J Clin Nutr. 2007;61(3):349–354. 16988647 10.1038/sj.ejcn.1602532

[11] Falony G, Honkala S, Runnel R, et al Long-term effect of erythritol on dental caries development during childhood: A posttreatment survival analysis. Caries Res. 2016;50(6):579–588. 27806364 10.1159/000450762

[12] Honkala S, Runnel R, Saag M, et al Effect of erythritol and xylitol on dental caries prevention in children. Caries Res. 2014;48(5):482–490. 24852946 10.1159/000358399

[13] Makinen KK, Saag M, Isotupa KP, et al Similarity of the effects of erythritol and xylitol on some risk factors of dental caries. Caries Res. 2005;39(3):207–215. 15914983 10.1159/000084800

[14] Runnel R, Makinen KK, Honkala S, et al Effect of three-year consumption of erythritol, xylitol and sorbitol candies on various plaque and salivary caries-related variables. J Dent. 2013;41(12):1236–1244. 24095985 10.1016/j.jdent.2013.09.007

[15] Silness J, Loe H Periodontal disease in pregnancy. Ii. Correlation between oral hygiene and periodontal condtion. Acta Odontol Scand. 1964;22:121–135. 14158464 10.3109/00016356408993968

[16] Socransky SS, Haffajee AD, Cugini MA, et al Microbial complexes in subgingival plaque. J Clin Periodontol. 1998;25(2):134–144. 9495612 10.1111/j.1600-051x.1998.tb02419.x

[17] Hashino E, Kuboniwa M, Alghamdi SA, et al Erythritol alters microstructure and metabolomic profiles of biofilm composed of *Streptococcus gordonii* and *Porphyromonas gingivalis*. Mol Oral Microbiol. 2013;28(6):435–451. 23890177 10.1111/omi.12037

[18] Van der Veen MH, Volgenant CM, Keijser B, et al Dynamics of red fluorescent dental plaque during experimental gingivitis-A cohort study. J Dent. 2016;48:71–76. 26921667 10.1016/j.jdent.2016.02.010

[19] Van der Weijden GA, Timmerman MF, Nijboer A, et al Comparison of different approaches to assess bleeding on probing as indicators of gingivitis. J Clin Periodontol. 1994;21(9):589–594. 7806674 10.1111/j.1600-051x.1994.tb00748.x

[20] Janus MM, Keijser BJ, Bikker FJ, et al In vitro phenotypic differentiation towards commensal and pathogenic oral biofilms. Biofouling. 2015;31(6):503–510. 26212722 10.1080/08927014.2015.1067887

[21] Exterkate RA, Crielaard W, ten Cate JM Different response to amine fluoride by *Streptococcus mutans* and polymicrobial biofilms in a novel high-throughput active attachment model. Caries Res. 2010;44(4):372–379. 20668379 10.1159/000316541

[22] Kaman WE, Galassi F, De Soet JJ, et al Highly specific protease-based approach for detection of *Porphyromonas gingivalis* in diagnosis of periodontitis. J Clin Microbiol. 2012;50(1):104–112. 22075590 10.1128/JCM.05313-11 PMC3256702

[23] Cummings RT, Salowe SP, Cunningham BR, et al A peptide-based fluorescence resonance energy transfer assay for *Bacillus anthracis* lethal factor protease. Proc Natl Acad Sci U S A. 2002;99(10):6603–6606. 11997440 10.1073/pnas.062171599 PMC124449

[24] Ciric L, Pratten J, Wilson M, et al Development of a novel multi-triplex qPCR method for the assessment of bacterial community structure in oral populations. Environ Microbiol Rep. 2010;2(6):770–774. 23766283 10.1111/j.1758-2229.2010.00183.x

[25] O’Donnell LE, Robertson D, Nile CJ, et al The oral microbiome of denture wearers is influenced by levels of natural dentition. PLoS One. 2015;10(9):e0137717. 26368937 10.1371/journal.pone.0137717 PMC4569385

[26] Koopman JE, Buijs MJ, Brandt BW, et al Nitrate and the origin of saliva influence composition and short chain fatty acid production of oral microcosms. Microb Ecol. 2016;72:479–492. 27155967 10.1007/s00248-016-0775-z PMC4937104

[27] Chen T, Yu W-H, Izard J, et al The human oral microbiome database: a web accessible resource for investigating oral microbe taxonomic and genomic information. Database (Oxford). 2010;2010:baq013. 20624719 10.1093/database/baq013 PMC2911848

[28] Ø H, Harper D, Ryan P PAST-PAlaeontological STatistics, ver. 1.89. Palaeontol Electron. 2001;4(1):1–9.

[29] Segata N, Izard J, Waldron L, et al Metagenomic biomarker discovery and explanation. Genome Biol. 2011;12(6):R60. 21702898 10.1186/gb-2011-12-6-r60 PMC3218848

[30] Söderling EM, Hietala-Lenkkeri A-M Xylitol and erythritol decrease adherence of polysaccharide-producing oral streptococci. Curr Microbiol. 2010;60(1):25–29. 19777305 10.1007/s00284-009-9496-6

[31] Abusleme L, Dupuy AK, Dutzan N, et al The subgingival microbiome in health and periodontitis and its relationship with community biomass and inflammation. Isme J. 2013;7(5):1016–1025. 23303375 10.1038/ismej.2012.174 PMC3635234

[32] Pozhitkov AE, Leroux BG, Randolph TW, et al Towards microbiome transplant as a therapy for periodontitis: an exploratory study of periodontitis microbial signature contrasted by oral health, caries and edentulism. BMC Oral Health. 2015;15:125. 26468081 10.1186/s12903-015-0109-4 PMC4607249

[33] Colombo AP, Boches SK, Cotton SL, et al Comparisons of subgingival microbial profiles of refractory periodontitis, severe periodontitis, and periodontal health using the human oral microbe identification microarray. J Periodontol. 2009;80(9):1421–1432. 19722792 10.1902/jop.2009.090185 PMC3627366

[34] Gonçalves LF, Fermiano D, Feres M, et al Levels of *Selenomonas* species in generalized aggressive periodontitis. J Periodontal Res. 2012;47(6):711–718. 22612405 10.1111/j.1600-0765.2012.01485.x PMC3678906

[35] Kumar PS, Griffen AL, Moeschberger ML, et al Identification of candidate periodontal pathogens and beneficial species by quantitative 16S clonal analysis. J Clin Microbiol. 2005;43(8):3944–3955. 16081935 10.1128/JCM.43.8.3944-3955.2005 PMC1233920

[36] Huang S, Yang F, Zeng X, et al Preliminary characterization of the oral microbiota of Chinese adults with and without gingivitis. BMC Oral Health. 2011;11:33. 22152152 10.1186/1472-6831-11-33 PMC3254127

