# Supplementary figures and images for: Effect of erythritol on microbial ecology of in vitro gingivitis biofilms
Source: J Oral Microbiol. 2017 Jun 22;9(1):1337477. doi: 10.1080/20002297.2017.1337477 (PMC5508376; doi:10.1080/20002297.2017.1337477)

A

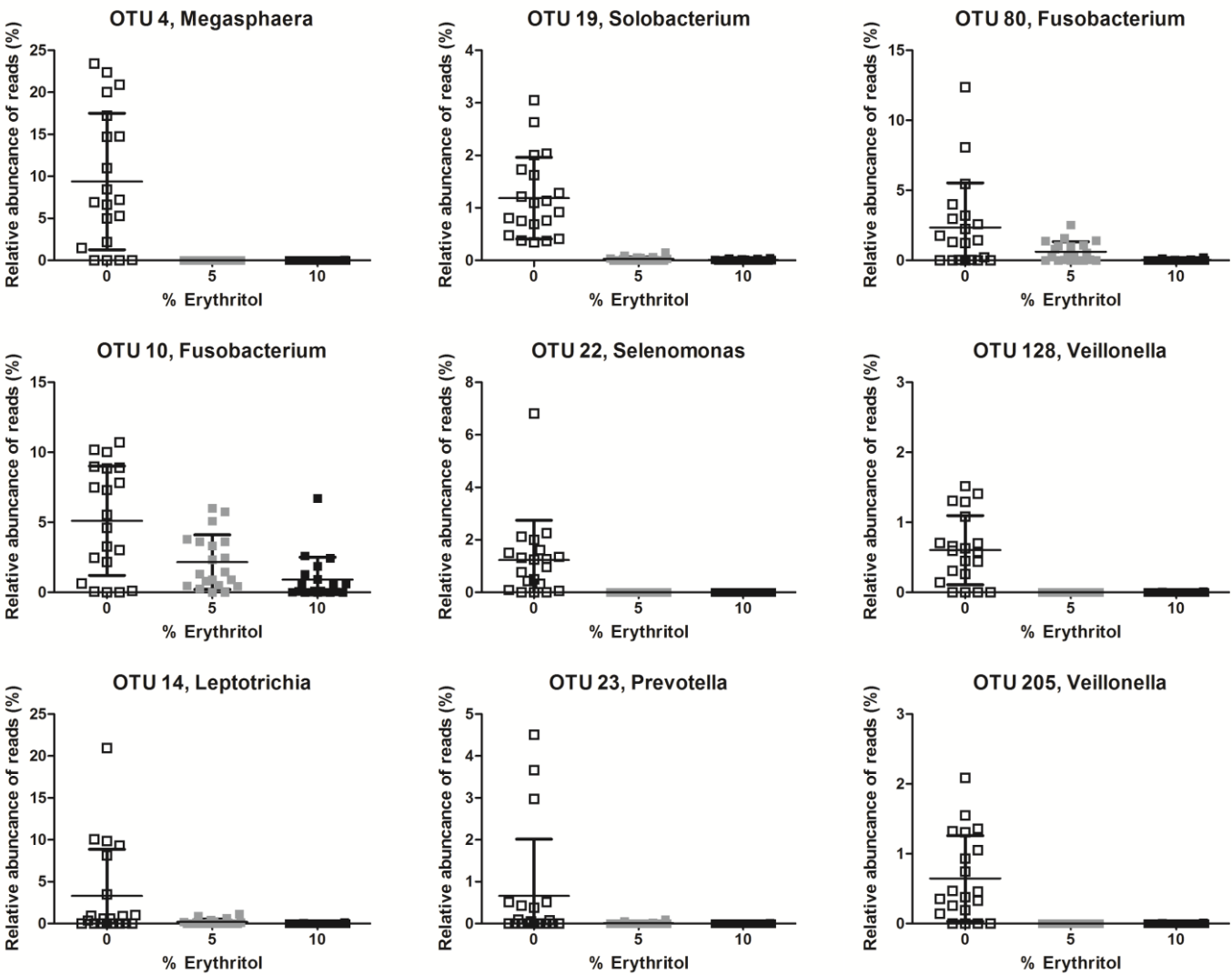

B

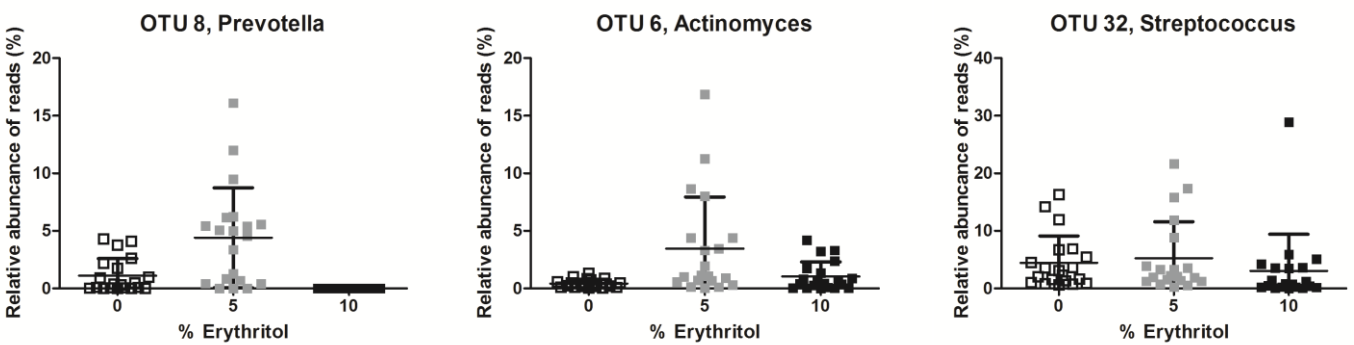

C

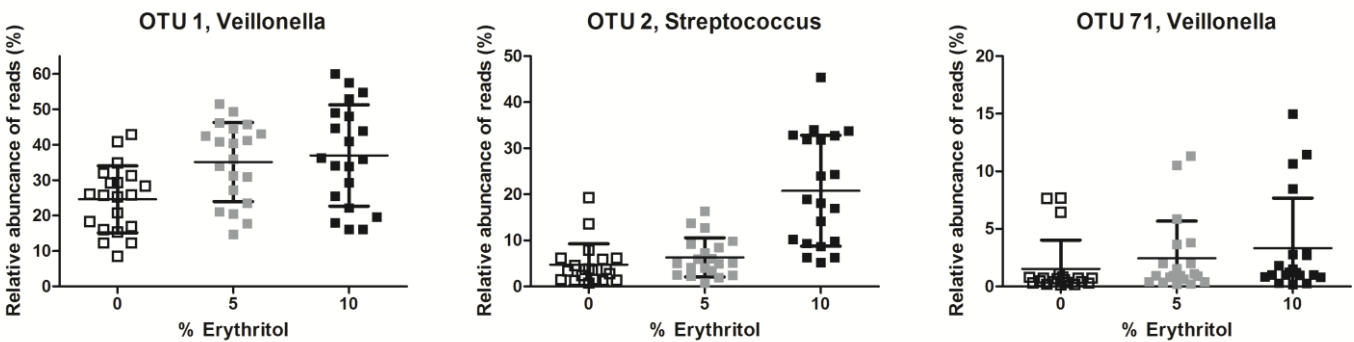

Supplement: Supplemental_data.zip [file zjom_a_1337477_sm7875.zip › Supplementary figure S1.pdf]
